# Resolution of reverse takotsubo cardiomyopathy secondary to presumed pheochromocytoma with intralesional hemorrhage

**DOI:** 10.1210/jcemcr/luag076

**Published:** 2026-04-18

**Authors:** Juan Cedeno-Serna, Raul Lopez Fanas, Girish Jayant, Christy Joseph, Daniel B Sims, David Carruthers

**Affiliations:** Department of Internal Medicine, Montefiore Medical Center, Bronx, NY 10467, USA; Division of Endocrinology, Diabetes and Metabolism, Department of Medicine, Montefiore Medical Center, Bronx, NY 10467, USA; Department of Internal Medicine, Montefiore Medical Center, Bronx, NY 10467, USA; Department of Internal Medicine, Montefiore Medical Center, Bronx, NY 10467, USA; Division of Cardiology, Department of Medicine, Montefiore Medical Center, Bronx, NY 10467, USA; Division of Endocrinology, Diabetes and Metabolism, Department of Medicine, Montefiore Medical Center, Bronx, NY 10467, USA

**Keywords:** stress cardiomyopathy, takotsubo syndrome, myocardial infarction, heart failure, pheochromocytoma, reverse takotsubo cardiomyopathy

## Abstract

A 38-year-old woman with a history of migraine headaches presented with episodic headaches, palpitations, chest pain, and worsening dyspnea. On arrival, she was hypertensive and tachycardic. Electrocardiography demonstrated inferolateral ST-segment depressions, QT interval prolongation, and elevated troponin levels, raising concern for acute myocardial infarction. Emergent coronary angiography revealed normal coronary arteries, while left ventriculography demonstrated basal hypokinesis with apical hyperkinesis, consistent with reverse takotsubo cardiomyopathy. Abdominal computed tomography identified a 3 cm right adrenal nodule, and 24-hour urine metanephrine levels were elevated to >8× the upper limit of normal, suggestive of pheochromocytoma. The patient was managed conservatively with alpha-adrenergic blockade. Follow-up abdominal magnetic resonance imaging demonstrated a decrease in nodule size to 2.3 cm with evidence of intralesional hemorrhage, and catecholamine levels subsequently normalized, leading to deferral of adrenalectomy. At 6-month follow-up, transthoracic echocardiography showed normalization of left ventricular function, and repeat abdominal computed tomography revealed marked reduction in the adrenal mass. At 1 year, she remains asymptomatic with normal biochemical testing.

## Introduction

Pheochromocytomas (PCCs) are a rare form of neuroendocrine tumor deriving from the chromaffin cells in the adrenal medulla that secrete catecholamines [1]. The annual incidence of PCC is ∼0.6 to 1.9 cases per 100 000 person-years [1]. About 25% to 40% of cases of PCC are related to hereditary syndromes, including pathogenic variants in *RET*, *VHL*, and *NF1* genes [2]. Clinical presentation often includes paroxysmal symptoms, such as palpitations, diaphoresis, headaches, and resistant hypertension [3]. Current guidelines recommend screening with serum or 24-hour urine fractionated metanephrines and normetanephrines to evaluate the presence of catecholamine excess, with levels >3× the upper limit of normal considered diagnostic in the absence of interfering substances or medications [4]. After catecholamine excess is established, adrenal protocol computed tomography (CT) scans of the abdomen and pelvis are recommended for tumor localization, and in some instances, magnetic resonance imaging (MRI) or functional imaging can be considered to evaluate for metastatic disease [2]. Pheochromocytoma can cause a variety of cardiovascular complications due to catecholamine excess, including arrhythmias, hypertensive crisis, myocardial infarction, cerebrovascular events, and, less commonly, stress-induced cardiomyopathy [5]. Takotsubo cardiomyopathy is a form of reversible stress-induced cardiomyopathy characterized by transient left ventricle dyskinesis beyond a single vascular distribution in the absence of obstructive coronary artery disease. It usually presents with apical ballooning [6], and less frequently can present with dyskinesis of the basal and midventricular segments with preserved apical contraction, known as reverse takotsubo cardiomyopathy (rTTC) [7]. If left untreated, PCC can result in serious complications, including cardiogenic shock, multiorgan failure, and cardiovascular collapse. Most cases of takotsubo cardiomyopathy are not associated with acute elevations of catecholamine levels apart from rare patients with PCC-induced takotsubo cardiomyopathy; however, there have been a few rare exceptions to this with takotsubo cardiomyopathy with elevated metanephrines, and pathology showed a benign adrenal adenoma [8, 9].

Standard management for PCC is 7 to 14 days of alpha-adrenergic blockade, followed by beta-adrenergic blockade, and followed by definitive management with preoperative volume expansion with high-salt diet and/or preoperative intravenous fluids and PCC surgical resection [10, 11]. Due to the high risk of complications, conservative and nonsurgical approaches are reserved for specific scenarios such as asymptomatic, nonsecreting, locally unresectable PCC, or advanced metastatic disease, and patients with high procedural risk [4]. However, spontaneous resolution of adrenal tumors due to bleeding or infarction has rarely been documented [12-14].

We present a unique case of a patient with suspected PCC who developed an unusual rTTC with subsequent spontaneous resolution after adrenal mass hemorrhage.

## Case presentation

A 38-year-old woman with a history of complex migraines and a known internal carotid artery aneurysm presented to the emergency department with palpitations, persistent retrosternal chest pain, sudden-onset dyspnea, nausea, vomiting, abdominal pain, dizziness, shakiness, and headache. She reported experiencing similar episodes over the past 2 years, typically self-resolving within 2 hours, but described a marked increase in their frequency during the preceding month, accompanied by weight loss of ∼20 pounds. She denied recent psychosocial stressors, any family history of cardiac or endocrine disorders, and use of any relevant substances or medications. On arrival, her vital signs were notable for hypertension (160/90 mmHg) and tachycardia (100 beats per minute).

## Diagnostic assessment

**Figure 1:**
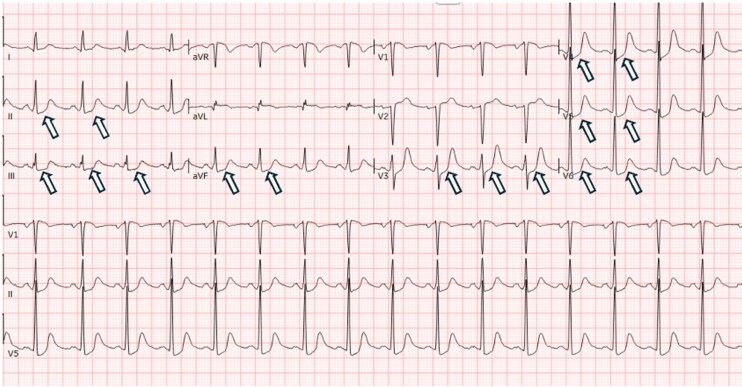
Initial electrocardiogram. ST depressions in II, III, AVF, and V4-V6 (white arrows) suggestive of inferolateral ischemia.

**Figure 2:**
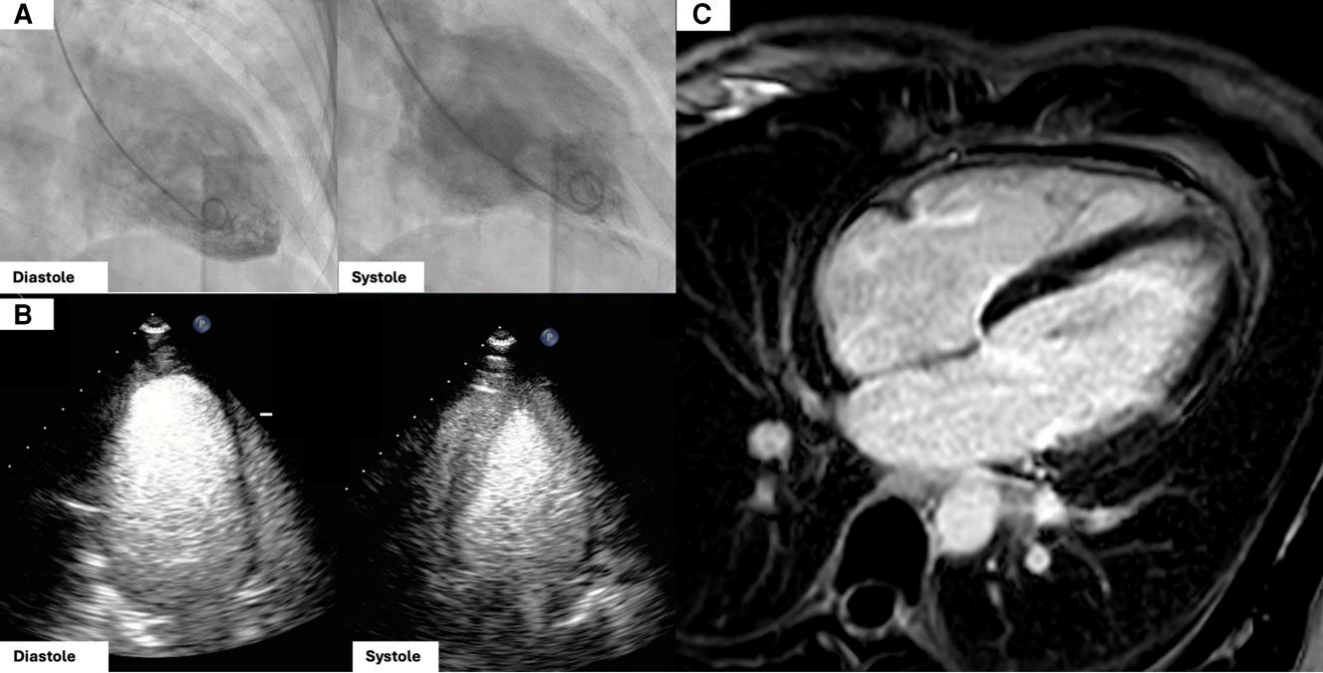
Initial cardiac imaging. (A) Ventriculography during diastole and systole with basal hypokinesis and conserved apical contraction. (B) Transthoracic echocardiogram in diastole and systole demonstrating anterolateral and inferolateral hypokinesis with preserved apical contraction. (C) Cardiac magnetic resonance demonstrating decreased resting perfusion in the subbasal endocardium and no late gadolinium enhancement.

Electrocardiography demonstrated inferolateral ST-segment depressions and QT-interval prolongation to 579 ms (Bazett corrected; Fig. 1). Serum troponin levels were elevated to 2.04 ng/mL (SI: 2.04 µg/L; reference range, <0.03 ng/mL [SI: <0.03 µg/L]; Table 1), raising initial concern for acute myocardial infarction. The patient underwent emergent left heart catheterization, which revealed angiographically normal coronary arteries. However, left ventriculography demonstrated basal hypokinesis with apical hyperkinesis, findings suggestive of rTTC (Fig. 2A). Subsequent transthoracic echocardiography (TTE) confirmed a mildly reduced left ventricular ejection fraction (45-50%) with anterolateral and inferolateral wall hypokinesis (Fig. 2B). Cardiac MRI revealed global left ventricular hypokinesis with relative apical sparing, decreased resting perfusion in the subbasal endocardium, and no late gadolinium enhancement (Fig. 2C), further supporting the diagnosis of rTTC.

**Table 1:** Laboratory findings obtained during the patient's hospital admission and follow-up

Test	At presentation	1 week from presentation	6-10 months from presentation	14 months from presentation	Reference range
Troponin*^a^*	2.04 ng/mL (2.04 µg/L)				<0.03 ng/mL (<0.03 µg/L)
Creatinine kinase	186 U/L (3.10 µkat/L)				<200 U/L (<3.33 µkat/L)
B-natriuretic peptide*^a^*	141 pg/mL (141 ng/L)				<100 pg/mL (<100 ng/L)
24-hour urine metanephrines	3364 µg/g cr (24 050 nmol/day)		134 µg/g cr (958 nmol/day)		32-134 µg/g cr (229-958 nmol/day)
24-hour urine normetanephrines	366 µg/g cr (2817 nmol/day)		142 µg/g cr (1093 nmol/day)		67-390 µg/g cr (516-3001 nmol/day)
24-hour urine total metanephrines	3730 µg/g cr (27 670 nmol/day)		276 µg/g cr (1973 nmol/day)		94-445 µg/g cr (672-3411 nmol/day)
Creatinine, 24-hour urine	1.41 g/day (12.46 mmol/day)				0.5-2.15 g/day (4.42-19.01 mmol/day)
Plasma renin activity	0.48 ng/mL/h (0.48 µg/L/h)				0.25–5.82 ng/mL/h (0.25-5.82 µg/L/h)
Metanephrine (MN), free		<25 pg/mL (<0.127 nmol/L)	<25 pg/mL (<0.127 nmol/L)	34 pg/mL (0.172 nmol/L)	≤57 pg/mL (≤0.289 nmol/L)
Normetanephrine (NMN), free		80 pg/mL (0.437 nmol/L)	43 pg/mL (0.235 nmol/L)	63 pg/mL (0.344 nmol/L)	≤148 pg/mL (≤0.808 nmol/L)
Metanephrine, total (MN + NMN)		80 pg/mL (0.4211 nmol/L)	43 pg/mL (0.2264 nmol/L)	97 pg/mL (0.570 nmol/L)	≤205 pg/mL (≤1.079 nmol/L)
Chromogranin A	104 ng/mL (104 µg/L)		<50 ng/mL (<50 µg/L)	50 ng/mL (<50 µg/L)	<311 ng/mL (<311 µg/L)
Aldosterone	8 ng/dL (222 pmol/L)				Supine 8:00-10:00 Am: 3-16 ng/dL (83-443 pmol/L)
Urine-free cortisol	12.7 µg/24 hours (35.0 nmol/day)				4.0-50.0 µg/24 hours (11.0-138.0 nmol/day)
Cortisol			8.5 µg/dL (234.6 nmol/L)		4.0-20.0 µg/dL (100.4-552 nmol/L)
Thyroid-stimulating hormone (TSH)	0.89 mIU/L		1.03 mIU/L		0.30-4.20 mIU/L
Dehydroepiandrosterone sulfate (DHEA-S)			109 µg/dL (2.96 µmol/L)		35-430 µg/dL (0.95-11.68 µmol/L)
Adrenocorticotropic hormone (ACTH)			8.9 pg/mL (1.958 pmol/L)		7.2-63.3 pg/mL (1.584-13.93 pmol/L)

^*a*^Values represent peak levels recorded.

**Figure 3:**
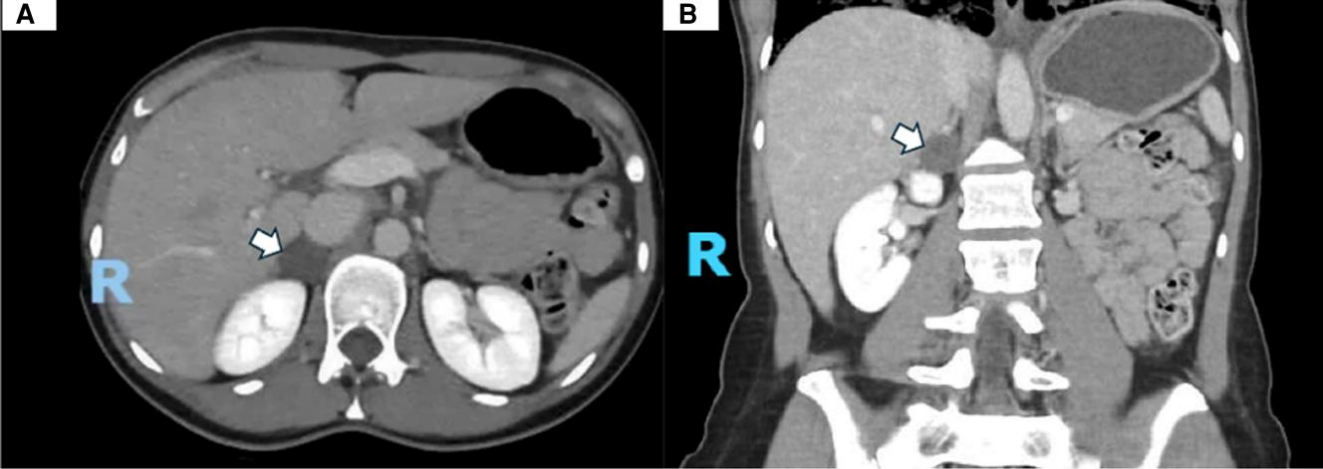
Computed tomography of the abdomen with intravenous contrast. The white arrows demonstrate a 3.0 × 2.2 × 2.6 cm well-circumscribed right adrenal nodular structure of 28 Hounsfield units in the axial view (A) and coronal view (B).

**Figure 4:**
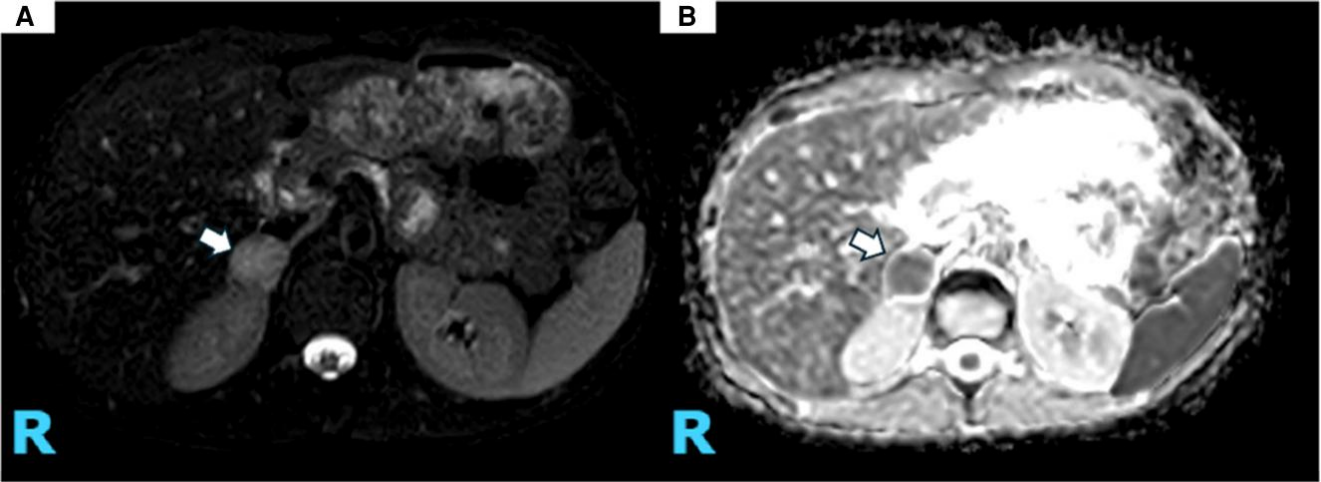
Magnetic resonance of the abdomen with contrast with adrenal gland protocol. (A) T_2_-weighted imaging demonstrates a 2.3 × 2.1 cm hyperintense nonenhancing adrenal lesion. (B) Restricted contrast diffusion within the lesion, suggestive of intralesional hemorrhage.

A 24-hour urine metanephrine collection revealed levels >8× the upper limit of normal (Table 1). Contrast-enhanced CT of the abdomen identified a right adrenal nodule measuring 3.0 × 2.2 × 2.6 cm with an attenuation of 28 Hounsfield units (Fig. 3), raising concern for PCC. A follow-up adrenal MRI performed 2 days later demonstrated restricted contrast diffusion within the lesion, suggestive of intralesional hemorrhage (Fig. 4).

## Treatment

The patient was initially treated with intravenous furosemide for pulmonary edema and subsequently started on lisinopril 5 mg daily and carvedilol 6.25 mg twice daily as guideline-directed medical therapy for heart failure. However, due to the concern for PCC, carvedilol was discontinued and replaced with doxazosin 1 mg daily for alpha-adrenergic blockade. Her symptoms improved significantly during hospitalization. The case was reviewed with the surgical team, who recommended multidisciplinary tumor board evaluation to determine surgical candidacy, with plans for repeat imaging and metanephrine testing. Low-dose metoprolol was added after adequate alpha-adrenergic blockade. The patient was discharged in stable condition.

## Outcome and follow-up

**Figure 5:**
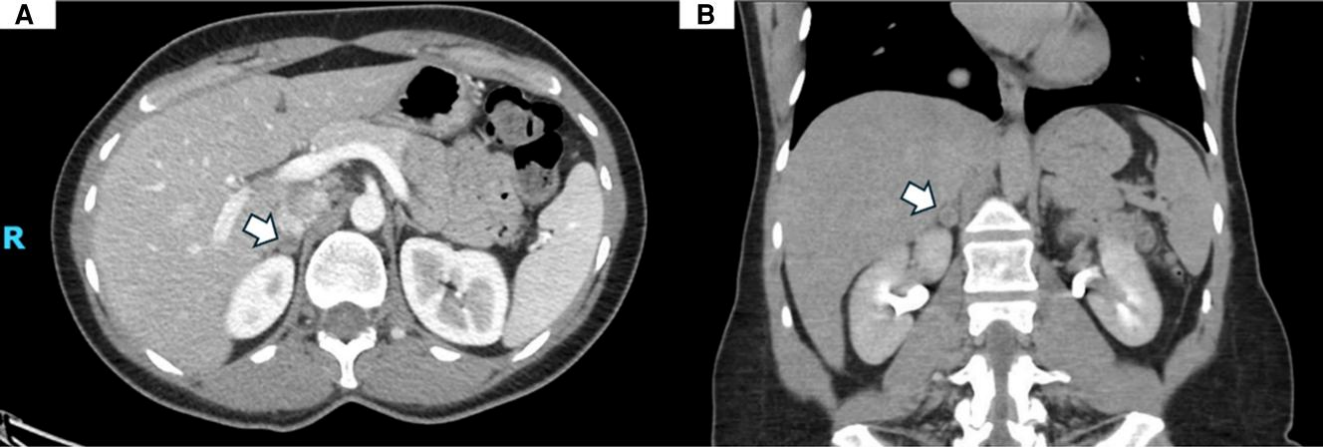
Repeat computed tomography of the abdomen with intravenous contrast at 1-year follow-up. The arrows show a 1.0 × 1.0 cm in the axial view (5A) and coronal view (5B) demonstrating an interval decrease of the adrenal lesion.

The patient remained asymptomatic, and within 1 week repeat serum metanephrines were within normal limits. The case was reviewed again in multidisciplinary tumor board. Given the atypical appearance for PCC on MRI, subsequent normalization of metanephrines, and resolution of symptoms, it was decided not to move forward with surgical intervention and instead follow-up with surveillance with periodic metanephrine measurement and adrenal imaging. A TTE performed 6 months after the index event demonstrated a normal left ventricular ejection fraction with no wall motion abnormalities. Eight months after the index event, a follow-up abdominal CT showed a decrease in the size of the right adrenal mass to 1 cm (Fig. 5); 24-hour urine metanephrine collections and repeat serum metanephrines remained within normal limits (Table 1). The patient was referred for genetic testing, which revealed no pathogenic variants. The patient was ultimately transitioned back to carvedilol, and lisinopril was discontinued. At 1-year follow-up, she remains clinically stable without recurrence of symptoms.

## Discussion

Stress-induced cardiomyopathy encompasses a spectrum of transient myocardial dysfunction syndromes, with takotsubo cardiomyopathy being increasingly recognized in recent years. Reverse TTC is a rare variant, characterized by basal hypokinesis with preserved or hyperkinetic apical segments, an imaging pattern that contrasts with the classic apical ballooning. While it represents only ∼2% of cases in the general population [15], its prevalence rises significantly in PCC-related presentations, where it accounts for up to 28% of cases, making it the second most common morphological pattern in this context [16].

Pheochromocytoma is a rare cause of stress cardiomyopathy, with most patients having normal catecholamine levels [8, 9]. The pathophysiology of stress cardiomyopathy secondary to PCC involves complex mechanisms that remain incompletely understood. It is known that catecholamine excess leads to overstimulation of beta-1 adrenoreceptors, increasing heart rate, contractility, and myocardial oxygen demand [17]. Concurrently, alpha-adrenoreceptor-mediated coronary vasoconstriction and microvascular dysfunction can exacerbate ischemia and trigger myocardial stunning [5]. Additional injury may result from oxidative stress, mitochondrial dysfunction, and endothelial impairment [18]. The catecholamine exposure long term can also result in beta-adrenoreceptor desensitization, especially at the apex where receptor density is typically higher, potentially explaining the predilection for apical involvement in classic takotsubo [5]. In contrast, rTTC may reflect a different distribution or sensitivity of adrenergic receptors in the basal segments, particularly in younger individuals, although no studies to date have addressed regional receptor variation by age [19, 20].

Takotsubo induced by PCC occurs more commonly in females and presents on average 18 years younger than patients with primary takotsubo [16, 21]. Patients under the age of 50 appear to experience higher rates of complications [16], and the reverse pattern is associated with worse outcomes than the classic form [21]. Complications occur in up to 68% of PCC-related cases, with heart failure (53%) and cardiogenic shock (37.7%) being the most common [15]. Current guidelines support early surgical resection, either laparoscopically or via open surgery [21-25]. Conservative management is not considered standard of care, as nonoperative approaches have been linked to high mortality and, in some cases, the need for cardiac transplantation [23-25].

A distinctive feature of our case was the presence of spontaneous adrenal hemorrhage, which preceded clinical improvement and normalization of catecholamine levels, which is a rare yet previously reported phenomenon. Up to 84% of spontaneous adrenal hemorrhages are associated with adrenal neoplasms, and PCC accounts for nearly half of them [13]. Very few cases in the literature have described resolution of PCC-induced cardiomyopathy following adrenal hemorrhage without surgical resection, and neither involved rTTC [10-12]. In cases such as these, it is essential to have a multidisciplinary specialty team review the case and for ongoing careful monitoring, as leaving an underlying neoplasm can carry a risk of recurrence and regrowth [25]. Accordingly, our patient will undergo annual plasma metanephrine measurements and yearly noncontrast CT imaging of the abdomen and pelvis to assess for interval growth. If no interval change in adrenal adenoma size or features and no elevation in serum metanephrines, we will consider discontinuation of yearly imaging after 2 to 3 years but recommend continued yearly metanephrine screening.

Our case has several atypical features. Firstly, our only prehemorrhage adrenal imaging is a CT with contrast, with the adrenal nodule appearing avascular and lipid rich, which is not typical in PCC. Secondly, we do not have a tissue diagnosis, and genetic testing is not positive. However, we believe the case supports PCC as the most likely diagnosis. Pheochromocytoma can have an atypical appearance on imaging [26, 27]. We also believe that the magnitude of metanephrine elevation remains a critical distinguishing feature of her case. There is an overlap in metanephrine elevations between hospitalized patients without PCC due to acute stress and those with the tumor; however, elevations exceeding 5× ULN have been found to achieve a specificity of 98% for PCC [28]. While not definitive, an elevation >8× ULN is highly suspicious for PCC. Another distinct aspect of our case was the normalization of plasma metanephrines within 1 week, which is uncommon. Nonetheless, similar cases have documented catecholamine levels returning to normal as early as 9 days after the event, and PCC biochemical cure has been documented 1 to 2 weeks postoperatively in patients after adrenalectomy [29, 30], supporting the biological plausibility of the timeline observed here.

Our case adds to this limited body of literature by highlighting a rare presentation of rTTC associated with hemorrhagic PCC with resolution of symptoms and normalization of metanephrines.

## Learning points

- Catecholamine-secreting tumors may initially present with rTTC, a potentially life-threatening condition that necessitates prompt, multidisciplinary evaluation.
- Acute management of suspected PCC in the setting of cardiomyopathy requires careful cardiovascular and hemodynamic monitoring, along with timely initiation of appropriate alpha-adrenergic blockade.
- Adrenal hemorrhage or infarction can be identified on adrenal imaging and, in rare instances, may lead to resolution of symptoms and normalization of metanephrine levels without surgery.

## Data Availability

Data sharing is not applicable to this article as no datasets were generated or analyzed during the current study.

## Abbreviations

CT: computed tomography
MRI: magnetic resonance imaging
PCC: pheochromocytoma
rTTC: reverse takotsubo cardiomyopathy

## References

[1] Saavedra T JS , Nati-Castillo HA, Valderrama Cometa LA, et al Pheochromocytoma: an updated scoping review from clinical presentation to management and treatment. Front Endocrinol (Lausanne). 2024;15:1433582. 39735644 10.3389/fendo.2024.1433582 PMC11671257

[2] Neumann HPH , Young WF, Eng C. Pheochromocytoma and paraganglioma. N Engl J Med. 2019;381(6):552‐565. 31390501 10.1056/NEJMra1806651

[3] Ando Y , Ono Y, Sano A, Fujita N, Ono S, Tanaka Y. Clinical characteristics and outcomes of pheochromocytoma crisis: a literature review of 200 cases. J Endocrinol Invest. 2022;45(12):2313‐2328. 35857218 10.1007/s40618-022-01868-6

[4] Shah MH , Goldner WS, Benson AB, et al Neuroendocrine and adrenal tumors, version 2.2021. J Natl Compr Canc Netw. 2021;19(7):839‐867. 34340212 10.6004/jnccn.2021.0032

[5] Szatko A , Glinicki P, Gietka-Czernel M. Pheochromocytoma/paraganglioma-associated cardiomyopathy. Front Endocrinol (Lausanne). 2023;14:1204851. 37522121 10.3389/fendo.2023.1204851 PMC10374018

[6] Templin C , Ghadri JR, Diekmann J, et al Clinical features and outcomes of takotsubo (stress) cardiomyopathy. N Engl J Med. 2015;373(10):929‐938. 26332547 10.1056/NEJMoa1406761

[7] Song BG , Chun WJ, Park YH, et al The clinical characteristics, laboratory parameters, electrocardiographic, and echocardiographic findings of reverse or inverted takotsubo cardiomyopathy: comparison with mid or apical variant. Clin Cardiol. 2011;34(11):693‐699. 22031226 10.1002/clc.20953 PMC6652294

[8] Y-Hassan S , Sörensson P, Ekenbäck C, et al Plasma catecholamine levels in the acute and subacute stages of takotsubo syndrome: results from the Stockholm myocardial infarction with normal coronaries 2 study. Clin Cardiol. 2021;44(11):1567‐1574. 34490898 10.1002/clc.23723 PMC8571561

[9] Cohen P , Aung H, Rayanagoudar G, Menon R. MON-395 Takotsubo cardiomyopathy and metanephrines. J Endocr Soc. 2019;3(Suppl 1):MON-395.

[10] Lenders JWM , Duh QY, Eisenhofer G, et al Pheochromocytoma and paraganglioma: an endocrine society clinical practice guideline. J Clin Endocrinol Metab. 2014;99(6):1915‐1942. 24893135 10.1210/jc.2014-1498

[11] Yip L , Duh QY, Wachtel H, et al American Association of Endocrine Surgeons guidelines for adrenalectomy: executive summary. JAMA Surg. 2022;157(10):870‐877. 35976622 10.1001/jamasurg.2022.3544 PMC9386598

[12] Takeshita Y , Teramura C, Takamura T. Vanishing of ruptured adrenal mass with takotsubo cardiomyopathy. Endocr J. 2018;65(12):1155‐1159. 30197382 10.1507/endocrj.EJ18-0119

[13] Marti JL , Millet J, Sosa JA. Spontaneous adrenal hemorrhage with associated masses: etiology and management in 6 cases and a review of 133 reported cases. World J Surg. 2012;36(1):75‐82. 22057755 10.1007/s00268-011-1338-6

[14] Atri A , Kanisha D, Nissa B. #1705577 “did my bleed cure me?”: pheochromocytoma that self-resolved with a spontaneous hemorrhage. Endocr Pract. 2024;30(5):11‐12.37805100

[15] Y-Hassan S . Clinical features and outcome of pheochromocytoma-induced takotsubo syndrome: analysis of 80 published cases. Am J Cardiol. 2016;117(11):1836‐1844. 27103159 10.1016/j.amjcard.2016.03.019

[16] Y-Hassan S , Falhammar H. Clinical features, complications, and outcomes of exogenous and endogenous catecholamine-triggered takotsubo syndrome: a systematic review and meta-analysis of 156 published cases. Clin Cardiol. 2020;43(5):459‐467. 32125009 10.1002/clc.23352 PMC7244299

[17] Costa VM , Carvalho F, Bastos ML, Carvalho RA, Carvalho M, Remião F. Contribution of catecholamine reactive intermediates and oxidative stress to the pathologic features of heart diseases. Curr Med Chem. 2011;18(15):2272‐2314. 21517751 10.2174/092986711795656081

[18] Akhtar MM , Cammann VL, Templin C, Ghadri JR, Lüscher TF. Takotsubo syndrome: getting closer to its causes. Cardiovasc Res. 2023;119(7):1480‐1494. 37183265 10.1093/cvr/cvad053

[19] Buijs EAB , Danser AHJ, Meijer NIF, Tibboel D. Cardiovascular catecholamine receptors in children: their significance in cardiac disease. J Cardiovasc Pharmacol. 2011;58(1):9‐19. 21654329 10.1097/FJC.0b013e31822233dd

[20] Brodde OE , Bruck H, Leineweber K, Seyfarth T. Presence, distribution and physiological function of adrenergic and muscarinic receptor subtypes in the human heart. Basic Res Cardiol. 2001;96(6):528‐538. 11770070 10.1007/s003950170003

[21] Agarwal V , Kant G, Hans N, Messerli FH. Takotsubo-like cardiomyopathy in pheochromocytoma. Int J Cardiol. 2011;153(3):241‐248. 21474192 10.1016/j.ijcard.2011.03.027

[22] De Angelis E , Bochaton T, Ammirati E, et al Pheochromocytoma-induced cardiogenic shock: a multicentre analysis of clinical profiles, management and outcomes. Int J Cardiol. 2023;383:82‐88. 37164293 10.1016/j.ijcard.2023.05.004

[23] Aw A , de Jong MC, Varghese S, Lee J, Foo R, Parameswaran R. A systematic cohort review of pheochromocytoma-induced typical versus atypical takotsubo cardiomyopathy. Int J Cardiol. 2023;371:287‐292. 36055473 10.1016/j.ijcard.2022.08.053

[24] Zhang R , Gupta D, Albert SG. Pheochromocytoma as a reversible cause of cardiomyopathy: analysis and review of the literature. Int J Cardiol. 2017;249:319‐323. 29121733 10.1016/j.ijcard.2017.07.014

[25] Li Z , Lai D, Jia Y, et al Predictors of postoperative recurrence of pheochromocytoma: a monocentric study. BMC Surg. 2025;25(1):179. 40281549 10.1186/s12893-025-02824-w PMC12023388

[26] Dogra P , Navin PJ, McKenzie TJ, et al Clinical, imaging and biochemical presentation of cystic pheochromocytomas. Clin Endocrinol (Oxf). 2023;98(1):32‐40. 35445428 10.1111/cen.14743 PMC9585148

[27] Blake MA , Kalra MK, Maher MM, et al Pheochromocytoma: an imaging chameleon. Radiographics. 2004;24(Suppl 1):S87‐S99. 15486252 10.1148/rg.24si045506

[28] Kline GA , Boyd J, Sadrzadeh HSM, Leung AA. Inpatient measurements of urine metanephrines are indistinguishable from pheochromocytoma: retrospective cohort study. Am J Med. 2021;134(8):1039‐1046.e3. 33864763 10.1016/j.amjmed.2021.03.015

[29] Narayanaswamy G , Sarma D, Saikia UK, Baro A, Bhuyan AK. Risk factors for perioperative complications, treatment outcomes and aggressive behavior of the tumor in patients with pheochromocytoma. J ASEAN Fed Endocr Soc. 2024;39(2):48‐53. 39620189 10.15605/jafes.039.02.07 PMC11604364

[30] Murai N , Azami T, Iida T, et al A case of pheochromocytoma with a marked decrease in catecholamine levels after rupture in which a good outcome was achieved by elective surgery. Endocr J. 2018;65(11):1093‐1099. 30078826 10.1507/endocrj.EJ18-0071

